# Rapid Screening for Entry Inhibitors of Highly Pathogenic Viruses under Low-Level Biocontainment

**DOI:** 10.1371/journal.pone.0030538

**Published:** 2012-03-02

**Authors:** Aparna Talekar, Antonello Pessi, Fraser Glickman, Uttara Sengupta, Thomas Briese, Michael A. Whitt, Cyrille Mathieu, Branka Horvat, Anne Moscona, Matteo Porotto

**Affiliations:** 1 Departments of Pediatrics, Microbiology and Immunology, Weill Medical College, Cornell University, New York, New York, United States of America; 2 PeptiPharma, Pomezia, Italy; 3 High Throughput Screening Resource Center, The Rockefeller University, New York, New York, United States of America; 4 Center for Infection and Immunity, Mailman School of Public Health, Columbia University, New York, New York, United States of America; 5 Department of Microbiology, Immunology and Biochemistry, Health Science Center, The University of Tennessee, Memphis, Tennessee, United States of America; 6 INSERM, U758, Ecole Normale Supérieure de Lyon, Lyon, France; 7 IFR128 BioSciences Lyon-Gerland Lyon-Sud, University of Lyon 1, Lyon, France; Virginia Polytechnic Institute and State University, United States of America

## Abstract

Emerging viruses including Nipah, Hendra, Lujo, and Junin viruses have enormous potential to spread rapidly. Nipah virus, after emerging as a zoonosis, has also evolved the capacity for human-to-human transmission. Most of the diseases caused by these pathogens are untreatable and require high biocontainment conditions. Universal methods for rapidly identifying and screening candidate antivirals are urgently needed. We have developed a modular antiviral platform strategy that relies on simple bioinformatic and genetic information about each pathogen. Central to this platform is the use of envelope glycoprotein cDNAs to establish multi-cycle replication systems under BSL2 conditions for viral pathogens that normally require BSL3 and BSL4 facilities. We generated monoclonal antibodies against Nipah G by cDNA immunization in rats, and we showed that these antibodies neutralize both Nipah and Hendra live viruses. We then used these effective Henipavirus inhibitors to validate our screening strategy. Our proposed strategy should contribute to the response capability for emerging infectious diseases, providing a way to initiate antiviral development immediately upon identifying novel viruses.

## Introduction

A continuous threat is posed by newly emerging and reemerging infectious diseases, many of which are of viral origin (reviewed in [1], [2]). Over the past decade, the global effort to meet this challenge has resulted in an enhanced ability to identify and genetically fingerprint the causative agent, often with extraordinary speed, as seen in the severe acute respiratory syndrome (SARS) episode in 2003–2004 [3] and the H1N1 swine influenza pandemic of 2009–2010 [4]. However, the speed at which we acquire genetic information on the causative agents of newly emerging infectious diseases is not matched by the speed at which we can develop suitable treatments. The genetic information in the episodes of SARS could not be translated into an equally rapid development of new therapies, since drug discovery, both by high-throughput screening (HTS) and rational design, requires information that does not easily derive from knowledge of the viral genome. Additionally, for novel emerging viruses, the resources required for classical drug discovery are not easily mobilized for diseases with limited market potential and/or sporadic outbreaks. However, these are exactly the situations where immediate availability of a specific, easy to use and HTS amenable system would be most valuable, since it would allow rapid testing of potential antiviral and immune activity.

For enveloped viruses, it is possible to identify the envelope glycoproteins directly from their genetic information, and to rapidly produce synthetic cDNAs corresponding to key domains of the viral fusion machinery. In this report, we outline a strategy that rapidly and predictably transforms these cDNAs into BSL2 amenable screening tools. We thereby identify a common screening platform applicable to multiple pathogens where the salient information (envelope glycoprotein cDNAs) can be identified by bioinformatic analysis of the viral genome. We can then screen for antiviral molecules that have high potency and acceptable pharmacological properties. Using a simple protocol for developing neutralizing antibodies and/or DNA vaccination, we validate the screening strategy and show that it can be used to screen for neutralizing antibodies from infected populations.

Nipah (NiV) and Hendra (HeV) viruses are two closely related, recently emerged, causative agents of zoonosis, capable of causing significant mortality in humans and animals [5], [6], [7]. Since their emergence (NiV in 1998 and HeV in 1994), both viruses have re-emerged several times with recent outbreaks showing, in the case of Nipah, well documented person-to-person transmission [8], [9], [10]. Almost every year since 2001, the virus has flared up in Bangladesh, killing 111 people in the last decade [1], [7], [11], [12]. There are no vaccines available for either virus, although both protein [13], [14] and DNA [15] vaccination approaches appear to be potentially effective. The alternative of passive immunotherapy has been shown to be effective in cat, hamster, and recently, ferret models of disease [14], [16], [17], [18]. However, both NiV and HeV are BSL4 agents, limiting the rapid development of antibodies and making large scale screening of antiviral compounds difficult [19].

The generation of monoclonal antibodies using cDNA immunization is highly valuable for rapid development of immunization strategies against a broad range of viruses, particularly in the case of new and emerging viruses. We show here that cDNA obtained from viral genomic information is sufficient to immunize animals and that this immunization elicits antibodies that are effective against live viruses. The cDNA can also be prepared directly from sequence and bioinformatic information about the viral glycoproteins, offering a quick route to passive immunization.

Key to the utility of the screening approach that we describe here is the use of the genes that encode envelope glycoproteins derived from a target virus to quickly assess potential antivirals. We transfect cells with plasmids that encode the target virus' envelope glycoproteins, and then infected the cells with vesicular stomatitis virus (VSV) lacking the gene for the entry glycoprotein G, but pseudotyped with VSV G. In this system we observed multi-cycle replication (MCR) of the target virus iedn the transfected cells [20]. We subsequently assessed antiviral agents for their ability to inhibit viral spread. This method has several advantages. It can be performed safely under BSL2 conditions, there is no need to produce pseudotyped viruses for each new emerging virus, and quantitative results can be obtained within 72 hours. We have successfully established this platform and demonstrated multi-cycle replication under BSL2 conditions for the 1918 influenza virus, a BSL3 pathogen, in addition to 3 BSL4 pathogens, Junin virus, NiV and the recently described Lujo virus [21], as proof of concept. The principle can be applied very easily to other viruses. We previously described a similar assay, that mimicked multicycle replication for HeV [20]. The new assay reported here however does not require a specific pseudotype to be produced for the primary infection, and hence will reduce the time required to set up an assay method tailored to each new emerging virus. The pseudotyped virions bearing the VSV-G required for the primary infection can be prepared in advance and in high titer. This will allow rapid screening of possible antiviral agents including antibodies, carried out in cells that reflect the natural host tissue. In addition, the screening assay can be adapted for immunological diagnostic analysis.

We envisage this pseudotyped MCR assay as a platform technology that will enable the preparation and storage of a specific sets of assay reagents for a wide range of viral pathogens, at low cost, *in advance* of any actual outbreak of the corresponding viral disease. This could form the basis for an efficacious and timely outbreak response, immediately following the identification of dangerous new viruses.

## Results

### A new multicycle viral replication assay enables assessment of high-risk viral pathogens under BSL2 conditions

**Figure 1:**
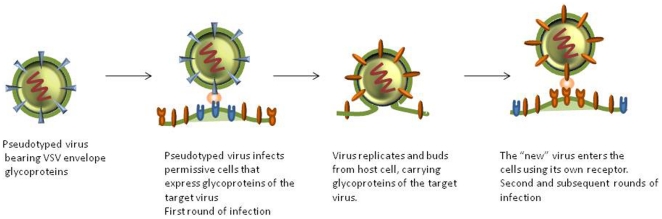
Modified multicycle viral replication assay. When VSV G pseudotyped viruses infect viral glycoprotein (G/F)-expressing permissive cells, multicycle replication is simulated where the initial entry is by VSV G pseudotyped virus but subsequent replication cycles are those of NiV pseudotyped virus produced after budding.

**Figure 2:**
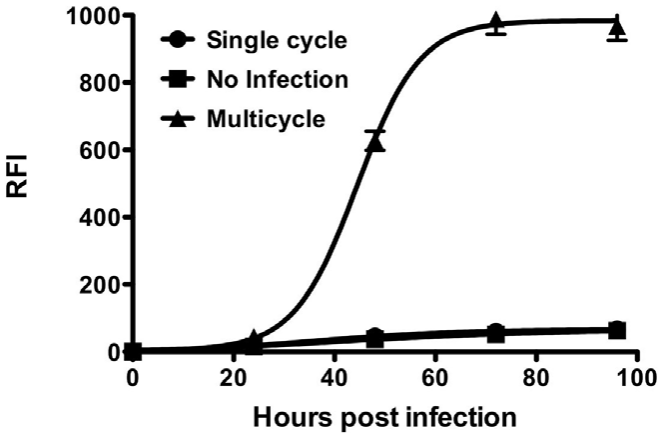
NiV multicycle replication using modified assay. Cells were transfected with plasmids encoding NiV G and F (triangles) and then infected with pseudotyped VSV. Transfected cells were also left uninfected as a control (squares). Additionally, control cells transfected with empty plasmid were also infected with pseudotyped VSV, showing single cycle replication (circles). Relative fluorescent intensities of the RFP were measured after 24, 48, 72 and 96 hours.

The multicycle replication assay that we described previously [20] as well as other BSL2 amenable assays [22], [23], [24], require production of pseudotyped stocks specific for each new emerging virus. This significantly adds to the time required to establish an assay for each new virus. In order to facilitate more universal application of this technology, we have established a simple system for multiple viruses that can be used for evaluating antivirals and antibody efficacy. We modified the original assay by using only a single pseudotyped virus for infection. The pseudotyped virus used in this system is prepared with a VSV background, using VSV delta G pseudotyped with VSV G. This enables virus entry mediated by VSV G, but does not result in subsequent rounds of infection by VSV. By supplying the envelope glycoproteins of the “new” virus in trans, the resulting virus production and release is of a pseudotyped “new” virus, which mimics native virus in terms of infection, replication and release (see Fig. 1). To test this concept, 293 T cells were transfected with plasmids encoding the surface glycoproteins of NiV (G/F), concomitantly with YFP (yellow fluorescent protein) to allow visualization of the transfection. Transfected cells were then either infected with pseudotyped VSV-ΔG carrying RFP (allowing visualization of infected cells) or left uninfected. A set of control wells with cells transfected with a control plasmid were also infected with the pseudotyped virus. The virus underwent multicycle replication in transfected cells as indicated by an increase in RFP expression over time after infection (Fig. 2). Even though the initial infection event is mediated by VSV pseudotyped virus, the second, and subsequent, rounds will be those of NiV due to the expression of the transfected glycoproteins (G/F). For high-throughput screening (HTS) a universal measure of assay quality and robustness, is the Z value. A Z′ value 1.0 is considered to be perfect, and assays with a value above 0.5 are required. The Z′ value for the multicycle replication as compared to the uninfected control was 0.83 (table 1), rendering it amenable to HTS.

**Table 1:** Z′ values of multicycle replication compared to no infection control using VSV pseudotype at 72 hours post infection.

Virus	Z′ value
Influenza	0.78
Junin	0.51
Lujo	0.84
NiV	0.72
VSV	0.83

### cDNA immunization of rabbits to generate polyclonal antibodies and inhibition of infection in the unmodified MCR assay

**Figure 3:**
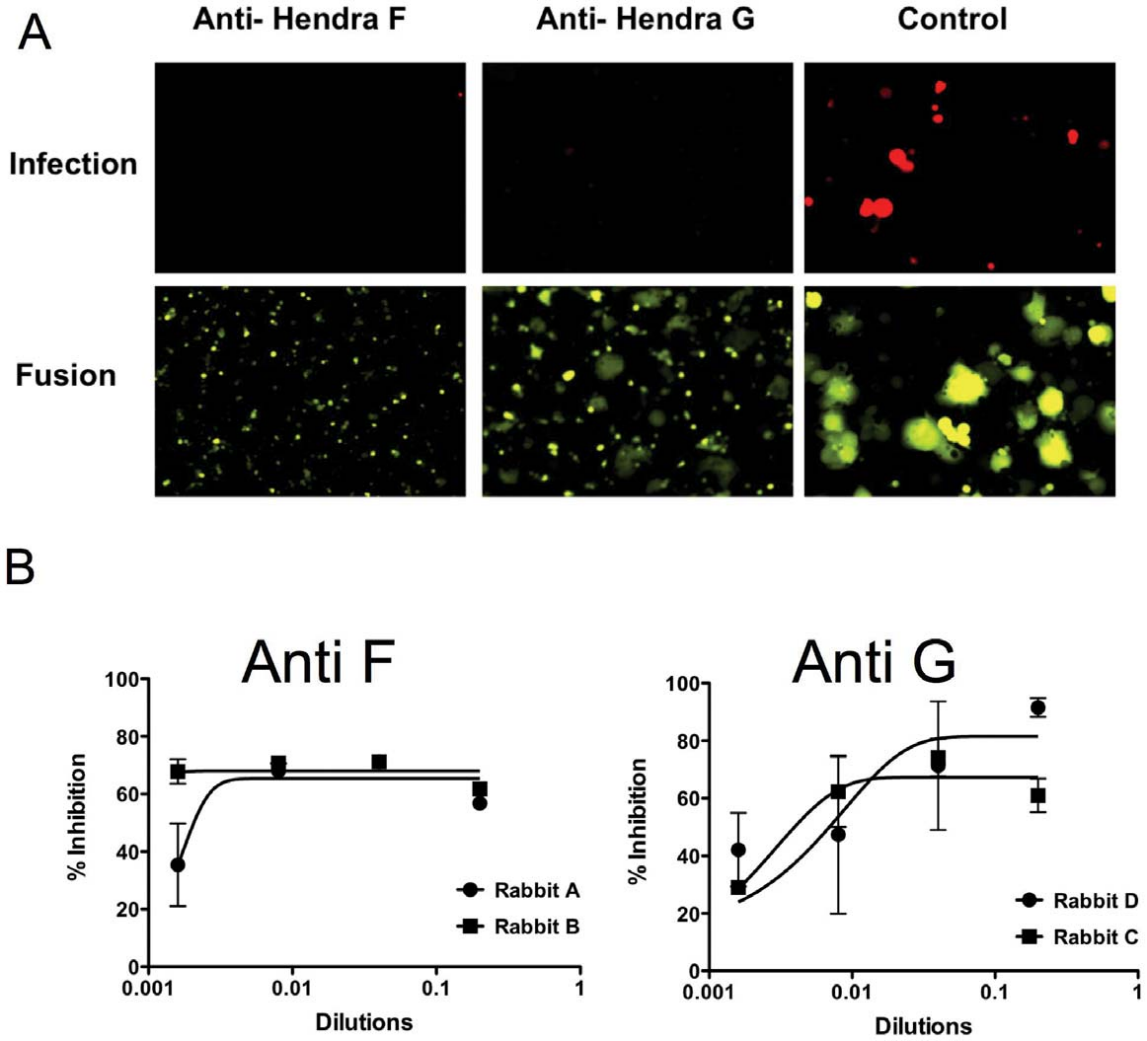
Multicycle replication (MCR) inhibition by antibody neutralization. (A) Cells coexpressing Nipah G/F and Venus-YFP were infected with pseudotyped VSV-ΔG-RFP-Nipah F/G, in the presence of anti-F or anti-G rabbit polyclonal antibodies. 48 hrs post-infection, the relative fluorescence intensities (RFI) were measured (B) and the spectral emission from the cells was converted into photographs (A). The bottom panel of photographs show extensive fusion in cells in the absence of antibodies (control), while no fusion is observed in the cells treated with anti-F antibodies, and some fusion is observed in the presence of anti-G antibodies. (B) polyclonal antibodies specific for HeV F or HeV G inhibit NiV pseudotype infection. Anti-HeV G antibodies show better dose response than anti-HeV F antibodies.

In parallel, we tested whether cDNA immunization would generate antibodies with high neutralizing activity and specificity. Rabbits were immunized with pCAGGS-HeV F or pCAGGS-HeV G. Serum was collected from the immunized animals and tested in the original (16) MCR assay format. Briefly, 293 T cells transfected with NiV F/G concomitantly with Venus-YFP were infected with pseudotyped NiV and infection was measured 48 hrs post infection by reading the intensity of RFP expression. Polyclonal antibodies from all 4 rabbits inhibited infection in our original pseudotyped viral entry assay format using NiV pseudotyped VSV (Fig. 3A). A small amount of cell-cell fusion can be seen in the presence of anti-HeV G antibodies while the anti-HeV F antibodies completely inhibit fusion. However, the anti-HeV G antibodies, especially from rabbit D, gave a better dose response than anti-HeV F antibodies (Fig. 3B).

### cDNA immunization of rats and evaluation of *in-vitro* neutralizing activity of anti-NiV G polyclonal and monoclonal antibodies using the new multicycle pseudotyped virus assay

**Figure 4:**
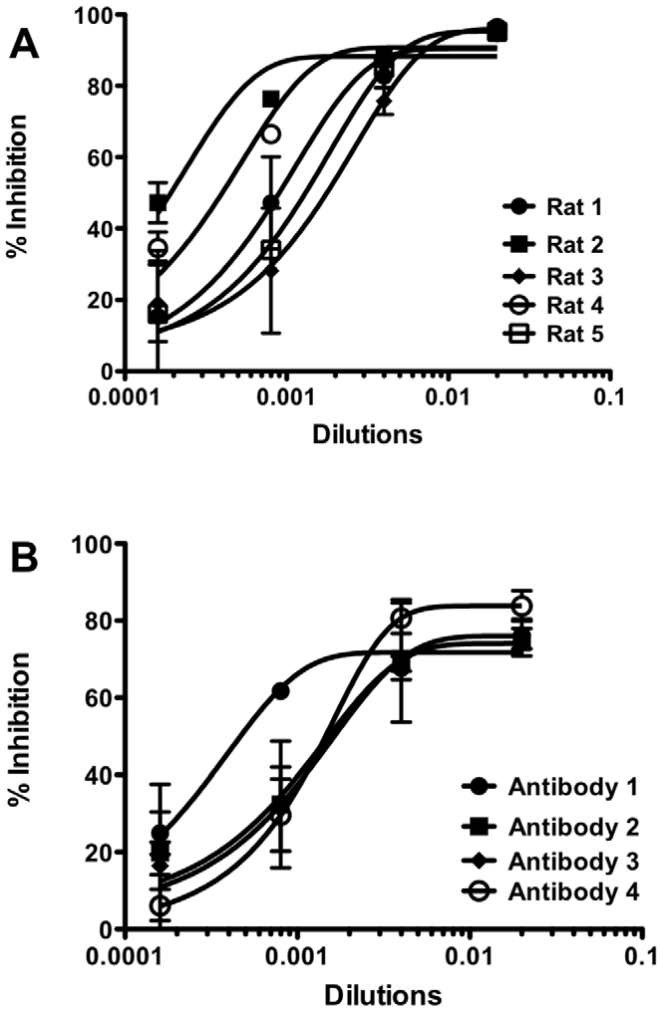
Inhibition of Multicycle replication by neutralizing antibodies. (A) Polyclonal antibodies were raised against NiV G in rats. While all the antibodies show inhibition of infection, the polyclonal antibodies from Rat #2 and Rat #4 show the strongest effect. (B) Anti-NiV monoclonal antibodies block NiV G/F in the MCR assay.

To test the universality of this approach, we immunized rats with cDNA to generate antibodies against NiV G. Five rats were immunized with pCAGGS-NiV G. As the first step, serum from five rats were collected and tested for inhibition of infection in the modified MCR assay. Infection was measured 48 hrs after infection. Polyclonal antibodies from three rats had relatively low titers of neutralizing activity. Polyclonal antibodies from Rat 2 and Rat 4 had much stronger neutralizing properties (Fig. 4A), showing greater inhibition at higher dilutions than Rats 1, 3 and 5. Monoclonal antibodies were also generated by immunizing rats with pCAGGS-NiV G, and tested in the MCR assay. At a concentration of 0.044 mg/ml, all monoclonal antibodies show 70%–80% inhibition of infection (Fig. 4B). However at a lower concentration of 0.009 mg/ml, only antibody 1 inhibits 60% of infection. Antibodies 2, 3 and 4 are able to inhibit only ∼40% of infection. The monoclonal antibodies were specific for NiV G by FACS analysis (data not shown).

### Validation of the cDNA-derived antibodies with authentic viruses

We considered the possibility that the cDNA immunization strategy may elicit antibodies that do not neutralize live viruses. The conformation of the proteins produced by the cDNA could be significantly different from that on the viral particles, resulting in induction of antibodies whose specificity could be significantly different from that induced during viral infection. For example, immunization with high concentrations of Ebola GP did not induce neutralizing antibodies [25]. In contrast, immunization with VLPs or VSV/Ebola GP chimeric virus induced neutralizing antibodies [26], [27]. To validate our strategy for live Nipah and Hendra viruses we performed a *in vitro* neutralization assay (Table 2). The neutralizing capacity of the polyclonal rabbit and monoclonal rat antibodies that we generated was compared to that of the murine mAb anti-NiV F, Gip21, previously reported to neutralize both NiV and HeV [18]. Although rabbit polyclonal anti-HeV G sera did not exhibit neutralizing capacity at the dilutions tested, both rabbit polyclonal anti-HeV F antibodies neutralized HeV, and one of these neutralized NiV infection as well. All rat monoclonal anti-NiV G antibodies inhibited NiV infection, and one of them neutralized HeV as well. Together, these results demonstrated that the immunization strategy applied in this study allows for generation of anti-viral polyclonal and monoclonal antibodies that neutralize live viruses.

**Table 2:** Antibody-mediated neutralization of live Nipah and Hendra viruses.

Antibody	Titer (NiV)*	Titer (HeV)*
Polyclonal anti-HeV G (rabbit #C)	<50	<50
Polyclonal anti-HeV G (rabbit #D)	<50	<50
Polyclonal anti-HeV F (rabbit #A)	800	1200
Polyclonal anti-HeV F (rabbit #B)	<50	300
mAb anti-NiV G (rat hybridoma 2)	600	<50
mAb anti-NiV G (rat hybridoma 1)	11400	200
mAb anti-NiV G (rat hybridoma 4)	2400	<50
Murine mAb anti-NiV F GIP21**	300	600

*Relative neutralization titer is presented as a reciprocal dilution of antibody samples that completely inhibited either NiV or HeV cytopathic effect.

**Control antibody [18].

### Validation of the new pseudotyped virus assay

**Figure 5:**
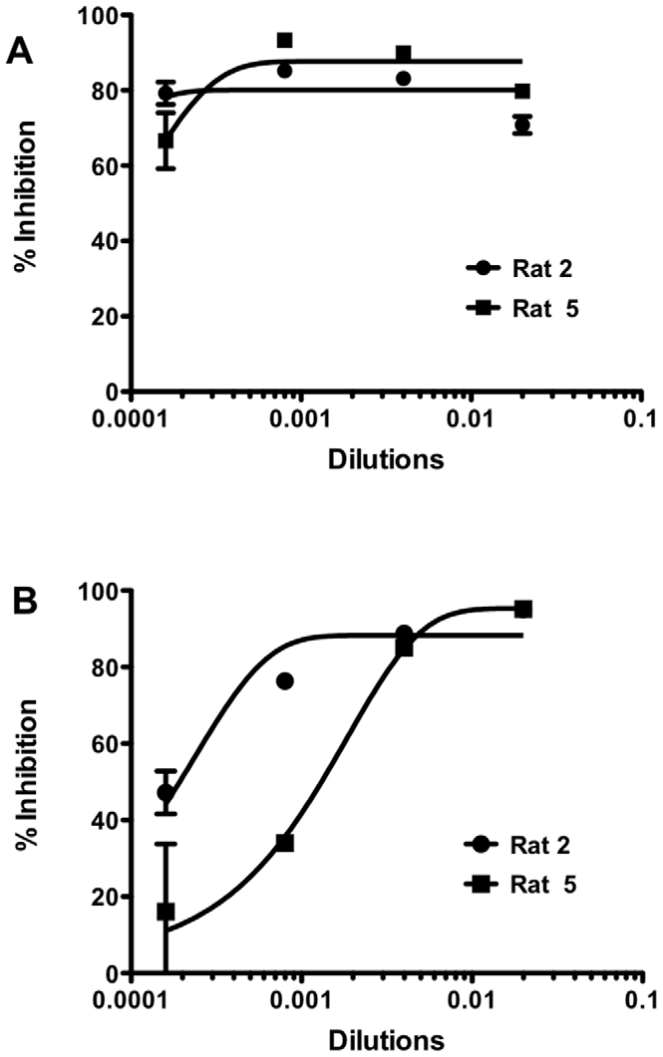
Validation of modified pseudotype assay validation for NiV. Cells were transfected with plasmids encoding the NiV glycoproteins F and G and then infected with either pseudotyped NiV (A) or pseudotyped VSV (B) in presence of rat anti-NiV antibodies, Rat 2 (circles) or Rat 5 (squares). Relative fluorescent intensities of the RFP were measured after 48 hrs.

To validate our modified pseudotyped virus assay, we compared the results of two rat polyclonal antibodies raised against NiV G using the assay described above and our modified MCR assay. These antibodies inhibited both the multicycle replication of NiV pseudotyped virus –the unmodified version of the multicycle replication assay, which uses the NiV pseudotype for infection (Fig. 5A)– and our newly modified assay described above using pseudotyped VSV for entry (Fig. 5B). However the effectiveness in the modified assay (Fig. 5B) was lower compared to the inhibition obtained when pseudotyped NiV was used for infection. For viral fusion inhibitors, we found that efficacy at inhibiting viral entry does not necessarily correlate with *in vivo* protection, while the ability of an inhibitor to block the spread of virus through a monolayer of cells after infection correlates positively with *in vivo* efficacy [28], [29]. Thus, this platform allows not only quick screening of antivirals, it may also help to accurately estimate the effectiveness of these compounds. Importantly, and perhaps explaining the apparently lower antiviral efficacy in the newly modified assay, this assay accounts for the context of viral spread, as distinct from merely the entry event. The ability to curtail multicycle replication even after infection has occurred is likely to provide an important advantage in the success of antiviral therapy.

### The modified MCR assay is adaptable to other enveloped viruses

**Figure 6:**
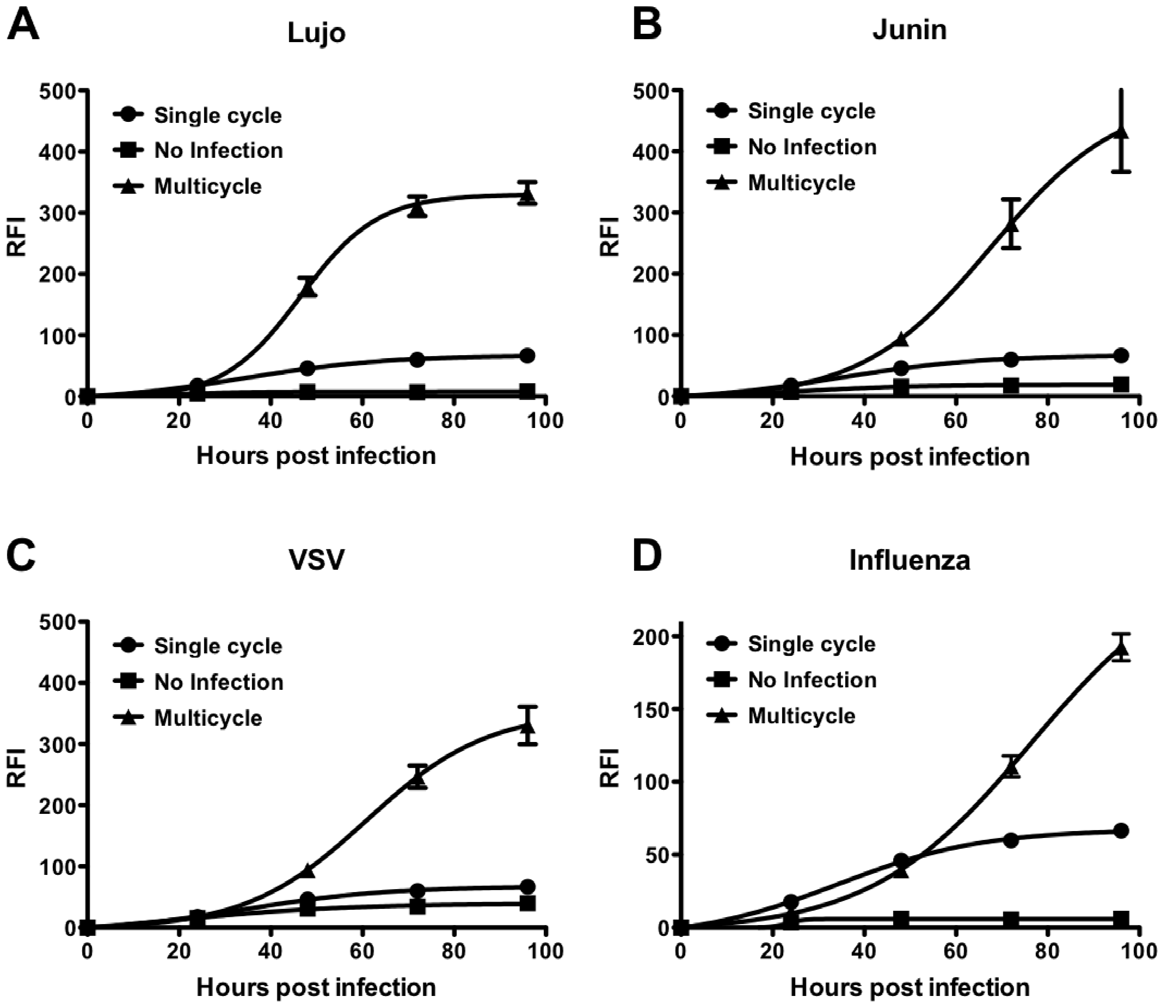
The modified assay can be adapted to other viruses. Cells were transfected with plasmids encoding glycoproteins from Lujo (A), Junin (B), VSV (C) or the influenza glycoprotein HA (D) then infected with pseudotyped VSV (triangles). Transfected cells were also left uninfected as a control (squares). Additionally, cells transfected with control empty plasmid were also infected with pseudotyped VSV showing single cycle replication (circles). Relative fluorescent intensities of the RFP were measured after 24, 48, 72 and 96 hours.

Once the assay was established, we investigated the application of this system to other viruses. For this purpose, 293 T cells were transfected with plasmids encoding the surface glycoproteins of Lujo virus (Fig. 6A), Junin virus (Fig. 6B) or VSV (Fig. 6C) concomitantly with YFP (yellow fluorescent protein) to allow visualization of the transfection. Transfected cells were then either infected with pseudotyped VSV-ΔG carrying RFP (allowing visualization of infected cells) or left uninfected. As an additional control, cells transfected with the control plasmid were also infected with the pseudotyped virus (single cycle). All the viruses underwent multicycle replication in cells transfected with the cDNA of the viral glycoprotein(s) as indicated by an increase in RFP expression over time post infection (Fig. 6). We observed minimal differences in RFP expression between cells during the initial round of infection (as expected) but as the length of incubation increased, and subsequent rounds of infection occurred in the multi-cycle system, RFP expression became 2–4 fold higher in transfected cells at 72 and 96 hours, respectively. This reveals a rapidly adaptible system that permits assessment of viral infection and antiviral efficacy for any enveloped virus. The Z′ values for the multicycle replication as compared to the uninfected controls were above 0.5 (table 1). This assay was performed in a 384 well plate format and showed very small deviations over large number of replicates.

To confirm that the modified assay(s) were useful, and specific for antiviral efficacy evaluation, we tested chloroquine (data not shown) – which we had previously shown to inhibit henipavirus multicycle replication –against the viruses in the MCR assay [20]. Chloroquine showed very high antiviral activity against NiV but very little activity against the other viruses at concentrations lower than 20 µM. When chloroquine was used at 100 µM it effectively blocked the VSV MCR assay as expected [30]. Note that chloroquine is a positive control for the cell based assay and it is not intended as a comparison for *in vivo* efficacy.

In addition to its utility for novel emerging viruses, this modified assay format can be applied to highly pathogenic influenza viruses that require a BSL3 facility. We adapted our MCR assay to evaluate replication of the 1918 influenza virus. We observed multicycle replication for this virus using TPCK treated trypsin treatment with neuraminidase treatment in the modified assay (Fig. 6D). The Z′ value for the multicycle replication compared to the uninfected control was 0.78.

## Discussion

Passive immunotherapy addresses the problem of quickly treating or protecting large populations of people against possible exposure to a virus, especially in the absence of a vaccine. Passive immunization is ideal for cases where the probability of exposure to infection is low or the causative agent is unidentified. However, quick generation of antibodies is key to an effective immunization plan, particularly in the case of emerging viruses. One problem that hinders the development of passive immunotherapy is the effective generation of antibodies. Traditionally, antibodies have been raised against either the dead/attenuated form of the causative agent, or against the entire or a subunit of a protein involved in the infection [13]. However, this method suffers from problems of purity and amount, though a high level of immunity is usually generated. cDNA immunization avoids the above problems since the viral glycoproteins are expressed on the cell surface in the animal, allowing the antibodies to be raised against the native form of the protein and thus to attain high activity and specificity [31], [32], [33]. This method is particularly suited for the generation of antibodies against membrane proteins. We show here that cDNA immunization of rats using cDNA corresponding to the surface glycoproteins of viruses, (NiV in our example), effectively generates monoclonal antibodies whose activity and specificity can be tested using a platform adaptable to a broad range of viruses.

An important advantage of the cDNA immunization method is that it rests on the sequence information of the viral genome, as in the case of envelope glycoproteins of unidentified infections, including zoonoses that have recently adapted to infection of humans. Often, transport of the infectious agent is not possible, limiting the development of antibodies, since the resources required for antibody generation may not be close to the site of viral detection. This can be especially true of emerging diseases, as highlighted by repeated NiV re-emergence in rural Bangladesh [34]. In these cases, cDNA immunization offers a fast and effective way to generate antibodies with specific neutralizing activity for the virus under investigation. Our results indicate that the antibodies raised by cDNA immunization neutralized live virus. Interestingly, several of the anti-F antibodies raised against Hendra F also blocked Nipah infection. While all the monoclonal anti-NiV G mAbs neutralized NiV, just one of them exhibited significant cross-neutralization against Hendra virus. Our data taken together with previously published reports [14], [16], [17], [18] may indicate that eliciting anti-F antibodies, at least for the cDNA immunization, may lead to more broadly neutralizing antibodies [18].

The use of passive immunotherapy has the advantage of generating immunity immediately in the patient. However, evaluating the neutralizing activity of potential therapeutic antibodies requires infection of cells with the virus of interest. This is a limiting problem in antibody generation when the virus is a BSL4 pathogen like NiV and HeV, or where there are difficulties associated with transportation of the virus from the point of detection to a suitable laboratory. The assay that we describe overcomes these problems by using pseudotyped viruses to infect cells that express glycoproteins of the same virus. Thus, the neutralizing activity of the antibody can be analyzed and evaluated using an assay that simulates multi-cycle replication of a virus under BSL2 conditions. This not only increases the speed of the development of effective antibody protection, it also significantly reduces testing costs. The MCR assay distinguishes between antibodies of differing neutralizing activity and specificity and replicates the antiviral selectivity seen with live viruses. Antibodies showing high inhibition at only low dilutions may be doing so due to non-specific interactions with the surface glycoproteins, while weak antibodies show low levels of infection inhibition.

One hurdle that we have not addressed in the current study relates to the possibility that monoclonal antibodies developed using cDNA immunization of rats have an associated risk of invoking an immune response and human anti-rat antibodies. Such events negate the intended therapeutic effect of the antibody. Fortunately, the task of humanization to arrive at products that are not recognized as antigens in the recipient is now relatively straight forward. In the next phase of this research, we plan to use cDNA immunization of mice genetically modified to produce humanized antibodies. A human monoclonal antibody against Nipah G has shown partial protection in the ferret animal model [16]. The antibody was identified by screening a human Fab library and soluble G protein [35]. The strategy described in this manuscript would allow for the direct identification of neutralizing human monoclonal antibodies, without intermediate steps.

We propose that the system we describe could be designed as a kit that includes the genetic material for the VSV-ΔG-RFP [36], ultimately requiring only the specific viral cDNA to be mixed together with the transfection mixture just before addition to the cells. A cost effective transfection reagent would be a major advantage for such a kit, and therefore we adapted our assay to use PEI [37] for transfection, bringing down the cost of the transfection reagent by several fold and rendering the system feasible at very low budget. The system that we propose has some limitations. We can screen only for antivirals that target entry, and not other steps in the viral life cycle, and we require viral envelope proteins that are compatible with VSV.

In summary, we propose a novel platform for screening of antiviral compounds and antibodies against newly emerging viruses. This assay can be established rapidly using just the sequence information of new and emerging viruses. The assay is highly reproducible and sensitive and can be performed in a BSL2 facility, providing a safe method for potentially highly pathogenic newly emerging viruses that otherwise require BSL3 or BSL4 containment facilities. The assay behaves consistently at low cell numbers, and thus allows miniaturization to a 384 well format, making it amenable to high-throughput screening. This concept can also be easily applied to primary cells, which may reveal different antiviral potencies from those in laboratory adapted monolayers [28], [29]. If used in comparison with the unmodified multicycle replication assay, this strategy also reveals differences between agents that inhibit entry, and agents that inhibit tissue spread of viruses.

## Materials and Methods

### Cells and virus

293 T (human kidney epithelial) and Vero (African green monkey kidney) cells were grown in Dulbecco's modified Eagle's medium (DMEM; Mediatech-Cellgro) supplemented with 10% fetal bovine serum and antibiotics at 37°C in 5% CO_2_.

Pseudotyped viruses were generated using VSV-ΔG-RFP, a recombinant VSV derived from the cDNA of VSV Indiana in which the G gene is replaced with the Ds-Red gene (RFP). Pseudotypes with NiV F and G were generated as described previously [38]. Briefly, 293 T cells were transfected with plasmid encoding VSV-G or NiVF/G. Five hours post-transfection, the dishes were washed and infected (multiplicity of infection [MOI] of 0.5) with VSV-ΔG-RFP complemented with VSV-G. Supernatant fluid containing pseudotyped virus (NiV F/G or VSV-G) was collected 18 h post-infection and stored at −80°C.

NiV isolated from the cerebrospinal fluid of a patient was received from Dr. K.B. Chua and Dr. S.K. Lam (University of Malaya, Kuala Lumpur, Malaysia). HeV was obtained from Graham Lloyd at the exotic virus bank at Porton Down, England. NiV and HeV stocks were prepared by infecting Vero-E6 cells as previously described [39], in the INSERM Jean Mérieux biosafety level 4 (BSL-4) laboratory in Lyon, France.

### Chemicals

Chloroquine diphosphate salt was obtained from MP Biomedicals (cat# 193919)

### Pseudotyped entry assay mimicking multicycle replication

As described previously [20], [38], NiVF/G glycoproteins were pseudotyped onto VSV-ΔG–RFP and the resulting pseudotyped viruses were used to infect NiV F/G-expressing cells at an MOI of 0.125 for simulation of multicycle replication. RFP production at 24, 48, 72 and 96 hr was analyzed on a microplate fluorescence reader (Spectramax M5). For detecting RFP expression levels, the wells were read by excitation at 535-nm and emission at 579-nm. For the detection of YFP expression, the wells were read by excitation at 510-nm and emission at 535-nm. For the modified multicycle replication assay, VSV G glycoprotein was pseudotyped onto VSV-ΔG–RFP and the resulting pseudotyped viruses were used to infect viral glycoprotein(s)-expressing cells for a simulation of multicycle replication. For single-cycle infection assays, the VSV-G pseudotype was used at an MOI of 0.125 to infect 293 T cells transfected with control plasmid. For Influenza virus, 2.5 ug/ml of TPCK trypsin and 0.001 mu neuraminidase was added to the wells.

### Antibodies

Polyclonal antibodies were raised in rabbits (anti-HeV F or anti-HeV G) or rats (Anti-NiVG) by cDNA immunization with plasmids expressing either HeV G, HeV F or NiV G using standard, commercailly available, protocols (Genovac). Monoclonal antibodies were produced using proprietary protocols by Genovac, using the NiV G cDNA. Isotype of the mAbs used in this study was IgG2b.

### Virus neutralization assay

NiV and HeV neutralizing antibodies were tested using two-fold dilutions of samples as described previously [18].Diluted antibodies were incubated with virus (25 PFUs/well in 96-well microtiter plates) for 60 min at 37°C in DMEM (Gibco) supplemented with 2% FCS. A total of 2.5×10^4^ Vero cells were then added to each well as indicator cells, and 96-well plates were incubated for 4 days at 37°C. Relative neutralizing titers were defined as the reciprocal dilution of antibody samples that completely inhibited the cytopathic effect of either NiV or HeV.

### Plasmids and reagents

The genes encoding influenza HA 1918, Nipah G and F were commercially synthesized and then cloned into pCAGGS vector. Junin and Lujo GPC in pCAGGS from Dr. Thomas Briese.

### Testing of anti-NiV G antibodies

293 T cells were transfected with plasmids encoding NiV G/F and YFP. Four hours post- transfection, antibodies were added at the indicated dilutions and then the cells were infected with VSV-G pseudotyped virus or with NiV F/G pseudotyped virus at an MOI of 0.125.

### Data Processing and Normalization

The Z′ values were used an assessment of quality [40], using the 16 values of the inhibitor and the blank for each microtiter plate as follows:1−(3*St.dev. inhibitor+3*St.dev. blank)/|Average inhibitor−Average blank|
